# Giant Paraovarian Cysts in an Adolescent Female Patient: A Case Report and Literature Review

**DOI:** 10.7759/cureus.92234

**Published:** 2025-09-13

**Authors:** George Mpourazanis, Magdalini Aliri, Petros Papalexis, Evridiki Anagnosti, Christina Efthimiadou, Rafailia-Stavroula Lekka, Michaela Papadatou, Theodora-Isavella Georgopoulou, Zoi Anastasiadi, Panagiotis Tsirkas, Andreas C Lazaris, Christos Akrivis

**Affiliations:** 1 Department of Obstetrics and Gynecology, General Hospital of Ioannina G. Hatzikosta, Ioannina, GRC; 2 Unit of Endocrinology, First Department of Internal Medicine, Laiko General Hospital, National and Kapodistrian University of Athens, Athens, GRC; 3 Faculty of Medicine, Trakia University, Stara Zagora, BGR; 4 First Department of Pathology, School of Medicine, National and Kapodistrian University of Athens, Athens, GRC

**Keywords:** adnexal mass lesion, benign cystic lesion, giant paraovarian cyst, minimally invasive laparoscopy, paraovarian cyst, paratubal cyst

## Abstract

Paraovarian cysts (POCs) are noncancerous growths that develop next to the ovary and fallopian tube. POCs are deemed to be significant when they surpass the threshold of 150 mm. Clinical signs and symptoms arise as a result of the pressure impact on nearby organs or due to associated complications. This case study highlights a 13-year-old adolescent female patient who presented with severe lower abdominal pain, nausea, and vomiting with a rare clinical presentation of bilateral giant POCs. A pelvic ultrasound demonstrated the presence of bilateral cystic lesions. Surgical excision was performed with a Pfannenstiel incision, and two large cystic lesions from both sides were extracted. One giant paraovarian on the left side was excised with dimensions of 21 cm, and the right giant POC was also excised with dimensions of 8.5 cm. Lower abdominal pain subsided after the operation, and the patient was symptom-free. Histopathological examination confirmed that the cyst had a thin fibrous wall and was lined with bland tubal or flattened epithelium, identifying it as a benign POC. Three months after the operation, the patient was doing well with no signs of recurrence.

## Introduction

Based on scientific studies, paraovarian cysts (POCs), sometimes referred to as paratubal cysts or Morgagni's hydatid cysts, comprise 10% of all adnexal masses [1]. POCs are typically benign, arise from the mesothelium, and can be remnants of paramesonephric (Mullerian) and mesonephric (Wolffian) ducts [2]. Paratubal cysts or POCs can vary in location, histological characteristics, and development, originating from peritoneal mesothelium or paramesonephric structures. Cysts can be located on the fallopian tube and are encased in an epithelium that secretes substances, contributing significantly to the formation of cysts [3]. While 97% of POCs are benign, there have been instances where borderline and malignant epithelial tumors have been found. POCs can develop at any point in a woman's life and may also be rarely seen in children, and only 4% occur in adolescents [4]. POCs typically do not cause symptoms but may present uncommon symptoms due to pressure on nearby organs or complications like lower abdominal pain, constipation, frequent urination, torsion, rupture, or hemorrhage [5].

The preferred imaging technique for examining the abdomen or pelvis is ultrasound (transabdominal, transvaginal, or transrectal) [5]. Pelvic ultrasonography, magnetic resonance imaging, and computed tomography scans are performed in a smaller group of cases with cystic lesions for assessing POCs, which may represent 5-20% of total adnexal cystic masses [6]. The average age of appearance of POCs, according to studies, has been shown to be between the ages of 30 and 40, with 4.25% in postmenopausal women and 4% in adolescents [7]. The study on laparoscopic evaluation revealed that 40% of patients had paratubal cysts, 60% had POCs, 67.7% had unilateral cysts, 15.3% had bilateral cysts, and 17% had multiple small cysts on one side [8]. POCs that exceed 30 mm in size should be surgically removed because of their ongoing growth and the potential for torsion. Smaller cysts can be drained, yet the likelihood of recurrence remains high. It is clear that the best method for managing large POCs is through surgical excision, but there is no agreement on whether to use laparoscopic or open surgery techniques [5].

We present a case involving a 13-year-old female patient who came to the gynecological emergency department with complaints of sudden lower abdominal pain. Bilateral paraovarian cystic lesions were revealed by pelvic abdominal ultrasonography. Open laparotomy was the treatment of cystic lesions. This research highlights the clinical and diagnostic approaches used to assess these cystic lesions. Considering the normal values of tumor markers and clinical presentation, it is a rare case of giant bilateral POCs, and that is why this case is worth describing.

## Case presentation

A 13-year-old Caucasian adolescent girl who had experienced acute lower abdominal pain lasting less than 24 hours was transported to the gynecological emergency department. The pain was acute at the onset. The condition was linked to nausea and two to four episodes of vomiting. The young patient reported that she had no bowel or urinary symptoms. Neither weakness nor anorexia was reported.

The patient's vital signs showed high blood pressure, a temperature of 37°C, a pulse rate of 70 beats per minute, a respiratory rate of 17 breaths per minute, and an oxygen saturation of 97%. Body mass index was estimated at 22.5 kg/m². Her medical background indicated that she lacked a noteworthy medical or surgical history. The gynecological history showed that menarche began at age 13, with menstrual cycles occurring every 27 days and lasting five to six days. There was no dysmenorrhea or irregular menstrual periods.

The general physical examination revealed nothing noteworthy. On abdominal examination, there was tenderness on deep palpation in the hypogastric region. There was no rebound tenderness, guarding, or abdominal rigidity, and bowel sounds were audible. There were no signs of peritoneal irritation. At palpation, a firm and painless palpable mass with restricted mobility was evident. Her laboratory results showed no evidence of inflammation or increased values of tumor markers (Table 1).

**Table 1:** Preoperative and postoperative laboratory results WBC: white blood cell; LYMPH: lymphocyte; HGB: hemoglobin; HCT: hematocrit; INR: international normalized ratio; aPTT: activated partial thromboplastin time; PLT: platelet; CRP: C-reactive protein; AST: aspartate transferase; ALT: alanine transaminase; GGT: gamma-glutamyl transferase; ALP: alkaline phosphatase; ALB: albumin; GLC: glucose; TPR: total protein; UA: uric acid; URE: urea; CRE: creatinine; K+: potassium; Na+: sodium; TSH: thyroid-stimulating hormone; FREE T4: free thyroxine; FREE T3: free triiodothyronine; CA 125: cancer antigen 125; CA 15-3: cancer antigen 15-3; CA 19-9: cancer antigen 19-9; CEA: carcinoembryonic antigen; AFP: alpha-fetoprotein; HCG urine: human chorionic gonadotropin urine

Parameter	Day 0 (admission and operation)	Day 1	Day 3 (exit day)	3-month follow-up	Reference values
WBC	5.28k/μL	12.39k/μL	9k/μL	6.25k/μL	4-11k/μL
Neutrophils	54.9%	75.4%	64%	60%	40-75%
LYMPH	33.9%	15.2%	17%	40%	20-45%
HBG	13.8 g/dL	12.5 g/dL	12 g/dL	14 g/dL	11.8-17.8 g/dL
HCT	40.6%	36.9%	37%	42%	36-52%
INR	1.00	0.88	-	-	0.8-1.2
aPTT	30 seconds	22.40 seconds	-	-	26-36 seconds
PLT	287k/μL	275k/μL	286k/μL	289k/μL	140-450k/μL
CRP	0.16 mg/dL	1 mg/dL	0.25 mg/dL	0.20 mg/dL	0-0.80 mg/dL
AST	39 U/L	20 U/L	44 U/L	24 U/L	5-33 U/L
ALT	33 IU/L	21 IU/L	55 IU/L	29 IU/L	5-32 IU/L
GGT	35 IU/L	15 IU/L	66 IU/L	30 IU/L	5-31 IU/L
ALP	50 IU/L	53 IU/L	59 IU/L	45 IU/L	35-125 IU/L
ALB	5.0 g/dL	3.5 g/dL	4.2 g/dL	3.8 g/dL	3.5-5.1 g/dL
GLC	86 mg/dL	70 mg/dL	90 mg/dL	110 mg/dL	70-115 mg/dL
TPR	7.7 g/dL	6.3 g/dL	6.8 g/dL	8 g/dL	6.2-8.4 g/dL
UA	3.6 mg/dL	2.5 mg/dL	2 mg/dL	4.6 mg/dL	2.3-6.1 mg/dL
URE	40 mg/dL	15 mg/dL	25 mg/dL	44 mg/dL	10-50 mg/dL
CRE	1.06 mg/dL	0.89 mg/dL	0.90 mg/dL	0.98 mg/dL	0.5-1.1 mg/dL
K+	3.8 mmol/L	3.5 mmol/L	3.8 mmol/L	5 mmol/L	3.5-5.1 mmol/L
Na+	139 mmol/L	140 mmol/L	134 mmol/L	140 mmol/L	136-146 mmol/L
TSH	0.84 μIU/mL	-	-	2 μIU/mL	0.35-4.94 μIU/mL
FREE T4	1.01 ng/dL	-	-	0.80 ng/dL	0.70-1.48 ng/dL
FREE T3	3.03 pg/mL	-	-	1.90 pg/mL	1.88-3.18 pg/mL
CEA	<1.73 ng/dL	-	-	-	0-5 ng/dL
CA 125	26.9 U/mL	-	12 U/mL (<35.0 U/mL)	-	0-35 U/mL
CA 15-3	17.2 U/mL	-	-	-	0-31.3 U/mL
CA 19-9	2.41 U/mL	-	-	-	0-37 U/mL
AFP	<2.00 ng/mL	-	-	-	0.89-8.78 ng/mL
HCG urine	Negative	-	-	-	Positive/negative

**Figure 1:**
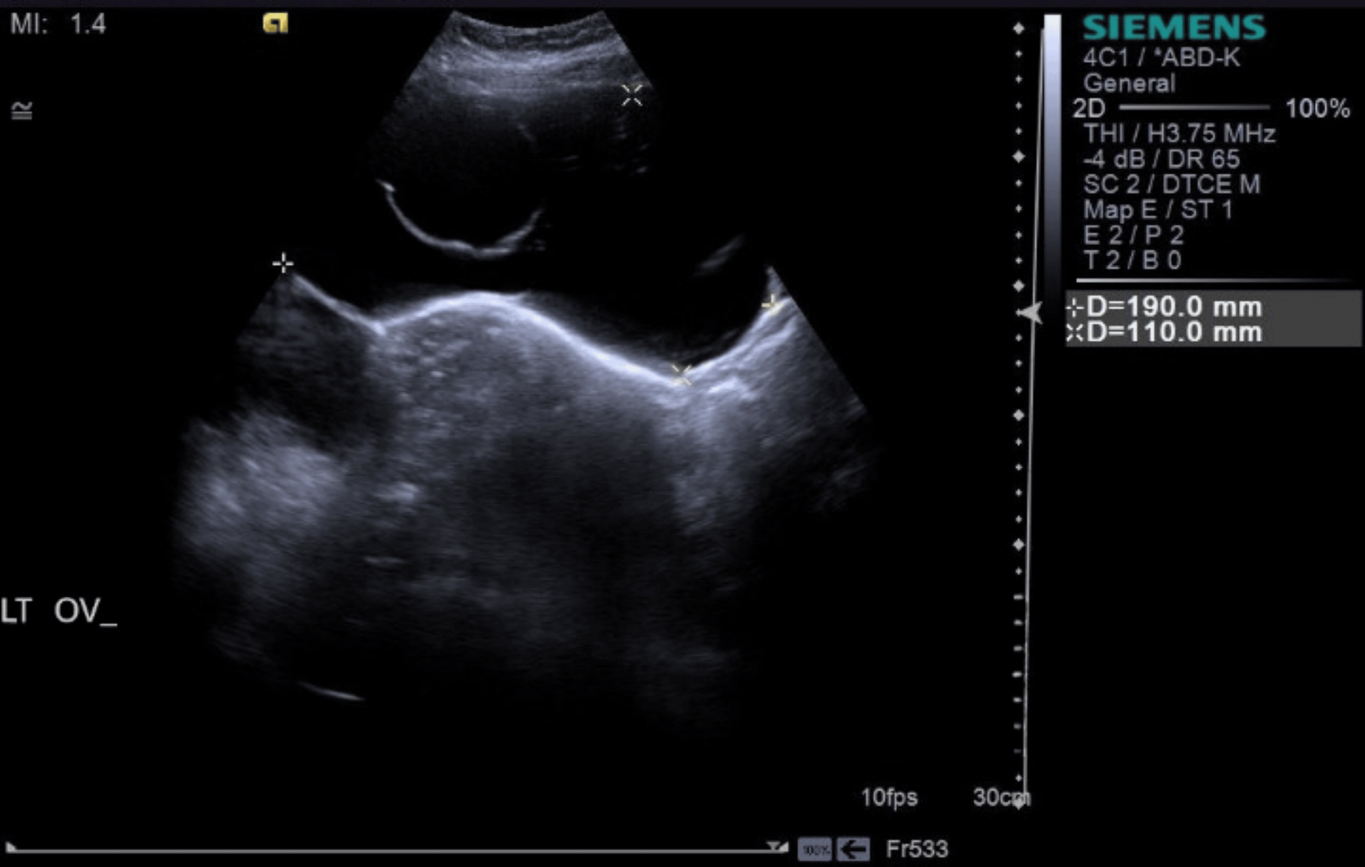
Pelvic ultrasonography showing a left ovary cyst with dimension 190.0 mm × 110.0 mm

**Figure 2:**
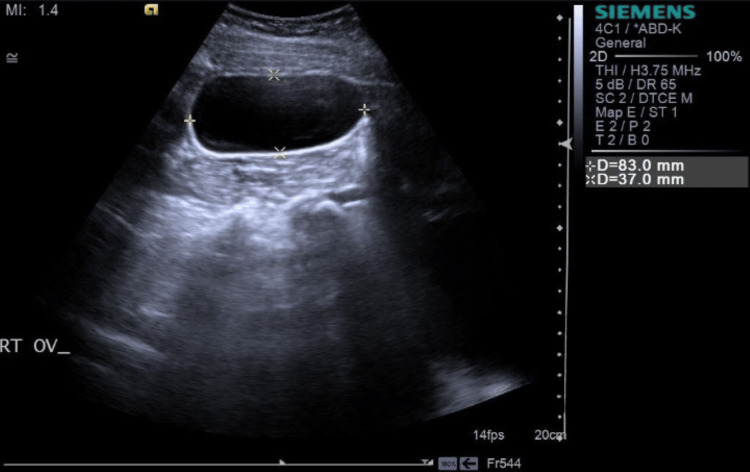
Pelvic ultrasonography showing a right ovary cyst with the dimension of 83.0 mm × 37.0 mm

According to pelvic abdominal ultrasonography, the anteverted uterus was of normal size, and both ovaries were of normal size. Bilateral cystic lesions were found; the right ovary cystic lesion measured 83.0 x 37.0 mm, while the left ovary cystic lesion measured 190.0 x 110.0 mm. Serous fluid was seen in both cystic lesions, and the enormous cystic lesion on the left ovary showed two fluid-filled chambers. The likelihood of ovarian torsion was reduced when a Doppler scan revealed normal color and spectral flow in both ovaries, including the cystic lesions (Figures 1, 2).

**Figure 3:**
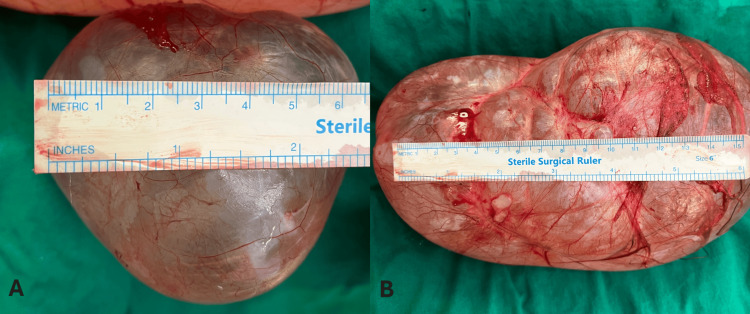
Macroscopic appearance of the excised paraovarian cysts. (A) Cystic formation surgically excised from the left ovary with a maximum diameter of 8.5 cm, with a smooth outer and inner surface. (B) Cystic formation surgically excised from the right ovary with a maximum diameter of 21 cm, with a smooth outer and inner surface

**Figure 4:**
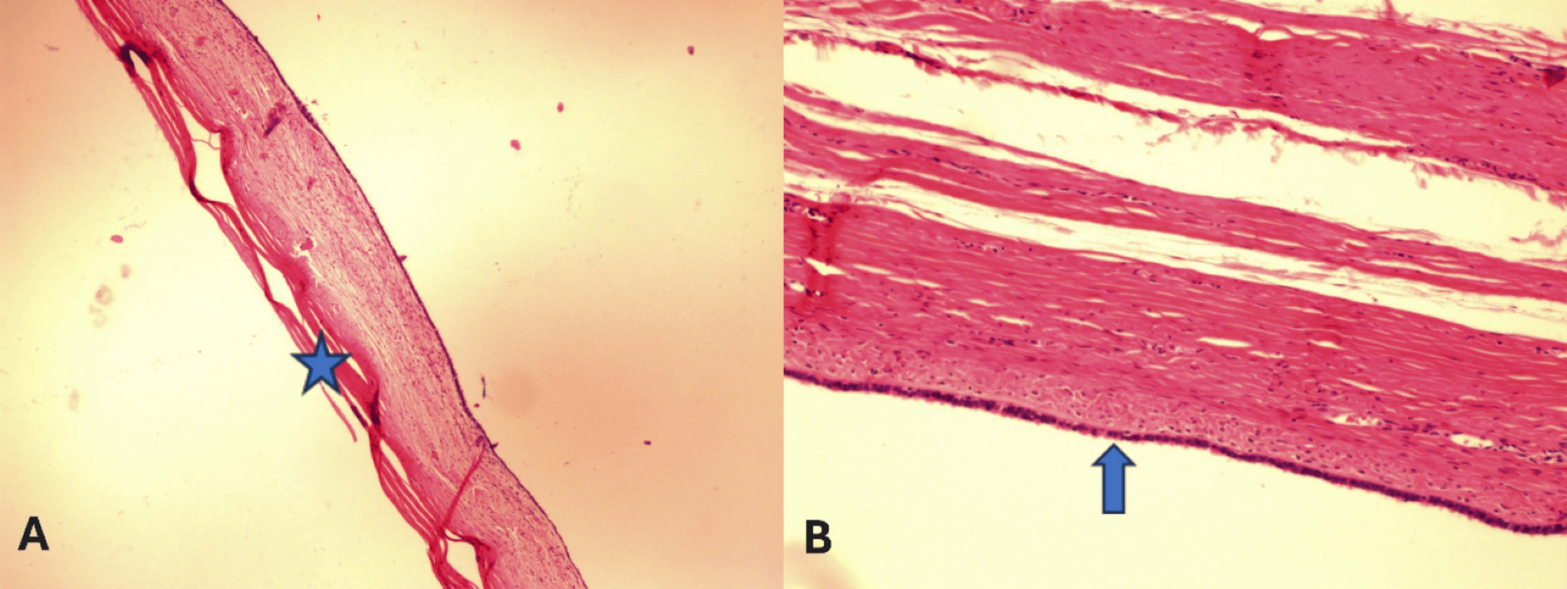
Histologic sections of the paraovarian cyst. (A) H&E stain 4×. (B) H&E stain 10×. This cyst has a thin fibrous wall (asterisk) and is lined by bland tubal or flattened epithelium (arrow) H&E: hematoxylin and eosin

After eliminating any other potential underlying condition with abdominal pelvic ultrasonography, a POC was considered. As a result of her intense pain, the patient was planned for an operation. In the operating room, the patient received general anesthesia and was placed in a supine position. A Pfannenstiel incision was performed, and we identified and located two large POCs. The uterus appeared normal, and no free fluid was detected in the pouch of Douglas. We surgically removed both cystic lesions, and we preserved both fallopian tubes and the ovaries. Hemostatic sutures were placed at the mesosalpinx between the ovary and the fallopian tube to ensure effective control of bleeding. Hemostasis was verified across the entire operative area. The abdominal wall layers were carefully repositioned in their proper anatomical sequence, and the closure of the skin was finalized with a continuous intradermal stitch. The left cyst specimen showed a maximum diameter of 21 cm, while the right cyst had a maximum diameter of 8.5 cm (Figure 3), and both were sent to the pathology department. Macroscopically, the cysts had a smooth surface. Upon incision, the cystic formations were filled with serous fluid. The cyst has a thin fibrous wall and is lined by bland tubal or flattened epithelium (Figure 4).

A final diagnosis of POCs was made. On the third day after surgery, the patient was discharged from the gynecology unit. There was no bilateral relapse of new cysts during the three-month follow-up.

## Discussion

POCs are typically benign, arise from the mesothelium, and can be remnants of paramesonephric (Mullerian) and mesonephric (Wolffian) ducts [2]. Research mentions that there is a possibility of the growth of POCs when the risk factor is obesity. POCs may contribute to infertility and ectopic pregnancies by affecting fallopian tube movement and constricting the passage [9]. Differential diagnosis in children and adolescents includes tubal and paratubal cysts, Mullerian abnormalities, tube-ovarian abscesses, infectious disorders, endometrioma, hydrosalpinx, pyosalpinx, and pregnancy-related masses [10].

A case study revealed that a minimally invasive procedure leads to improved clinical outcomes following surgery in a 17-year-old female patient [11]. A retrospective study involving 72 patients presenting with abdominal pain found that 41% experienced adnexal torsion as a complication and underwent a laparoscopic procedure. The findings indicate that laparoscopic removal of POCs is a viable treatment option for children [12]. Gadre et al. reported a case involving a patient with bilateral POCs measuring 50 x 40 cm. The individual underwent an exploratory laparotomy, which highlighted the importance of accurately diagnosing giant POCs in the context of a significant abdominal mass, as this condition is associated with tubal involvement and affects the patient's reproductive potential [13]. We observed a decrease in the levels of the cancer biomarker cancer antigen (CA) 125 after surgical removal of the giant POCs bilaterally in our case. This probably indicates that the biomarker CA 125 can act as a disease monitoring biomarker in such clinical cases.

Belouad et al. demonstrated that ultrasound examination may not always be practical for assessing giant POCs, making laparoscopy the most effective surgical procedure for treating these large cysts [14]. A 19-year-old female patient experiencing dysmenorrhea and lower abdominal pain underwent a diagnostic laparoscopy. The findings from this case indicated that timely surgical intervention can avoid complications such as fallopian tube necrosis, gangrene, and the need for tube removal, particularly in women of reproductive age, along with its long-term consequences [15].

Romeo et al. conducted a case study involving a 15-year-old patient, indicating that laparoscopy is favored over fertility-preserving surgery for large paratubal-POCs [16]. A retrospective review involving 51 patients with an average age of 31.8, all presenting with abdominal pain, revealed the presence of cystic lesions in both ovaries, with cyst sizes ranging from 7 to 18 cm. The analysis indicated that complications included cyst enlargement in 72.62% of cases, adnexal torsion in 18.51%, cyst rupture in 1.85%, hemorrhage in 7.4%, and benign tumors in 12.96%. All participants underwent laparoscopic surgery. The findings of this study suggested that the laparoscopic procedure resulted in a fertility rate of 57.39% [17].

It is referred to in the literature that there are no definitive guidelines available for managing POCs, even though they are quite common [5]. Casarin et al. found in their research that regardless of the size of the mass, laparoscopy should consistently be regarded as the optimal surgical method for treating benign adnexal masses [18]. In certain facilities that have limited resources and fewer skilled staff, open surgery remains an option for patients with POCs, as illustrated by the case we discuss. Nowadays, laparoscopic surgery is typically carried out in facilities with specialized practitioners and teams, leading to reduced trauma for the patient and improved results. All reported studies are mentioned in Table 2.

**Table 2:** Reported relevant studies on paraovarian cysts RT: right; LT: left

Study	Type of study	Number of patients	Age (years)	Symptoms	Cyst location	Imaging methods	Size of cyst (cm)	Surgical management	Follow-up after operation (months)	Complications	Pathology report	Results
Dechen et al. [11]	Case report	1	17	Abdominal pain	RT side	Computed tomography scan	23 × 13 cm	Exploratory laparotomy	1	Not mentioned	Benign paraovarian cyst	Minimally invasive surgery is the best choice for operation
Fryczek et al. [12]	Retrospective analysis	72	15	Abdominal pain	Not mentioned	Ultrasonography and computed tomography scan	1.8-32 cm	Laparoscopy	Not mentioned	Adnexal torsion (41%)	Paraovarian cyst	Laparoscopic excision is a safe, less invasive, and feasible treatment method for children
Gadre et al. [13]	Case report	1	26	Abdominal and pelvic heaviness	Bilateral	Ultrasonography	50 × 45 cm	Exploratory laparotomy	Not mentioned	Not mentioned	Benign serous cystadenoma	Giant paraovarian cyst diagnosis is crucial when dealing with a large abdominal mass, as it is linked to tubal involvement and the patient's reproductive outcomes
Belouad et al. [14]	Case report and review of the literature	1	16	Abdominal pain	RT side	Abdominopelvic ultrasonography	26 cm	Suprapubic mini-laparotomy	Not mentioned	Not mentioned	Paraovarian cyst	Ultrasound diagnosis for cysts is not always feasible. Laparoscopy is the better surgical operation for giant cysts
Syed et al. [15]	Case report	1	19	Dysmenorrhea and lower abdominal pain	LT side	Ultrasonography and computed tomography scan	3 cm	Laparoscopy	Not mentioned	Fallopian tube torsion	Paraovarian cyst	Timely surgical intervention can prevent complications like fallopian tube necrosis, gangrene, and tube removal, especially in reproductive-age women, and its long-term implications
Romeo et al. [16]	Case report and review of the literature	1	15	Abdominal pain	LT side	Ultrasonography and computed tomography scan	30 × 24 cm	Laparoscopy	6	Grade IV hydronephrosis	Paraovarian cyst	Laparoscopy is preferred over fertility-sparing surgery for giant paratubal-paraovarian cysts
Durairaj and Gandhiraman [17]	Retrospective analysis	51	31.8	Abdominal pain	RT side: 29; LT side: 19; bilateral: 3	Ultrasonography, computed tomography scan, and magnetic resonance imaging	7-18 cm	Laparoscopy	Not mentioned	Cyst enlargement (79.62%), adnexal torsion (18.51%), hemorrhage (7.4%), rupture (1.85%), and benign tumor (12.96%)	Serous cystadenoma and serous cystadenofibroma	Laparoscopy can attain fertility at 57.39%

In our research, histopathology findings indicated the presence of bilateral giant POCs without any indications of borderline or malignant changes, and there was no sign of recurrence after a three-month follow-up period. Additional clinical and laboratory studies are required in the future to better comprehend the precise mechanisms behind the formation and causes of these giant cystic lesions.

## Conclusions

Giant bilateral POCs are rare, especially in adolescents, and can cause sudden symptoms requiring immediate surgical treatment. Diagnosis is challenging, especially when comparing cysts to other adnexal masses. Open laparotomy can be effective in cases of limited laparoscopic capabilities. Open laparotomy continues nowadays to be a reliable and efficient therapeutic option when required, according to the clinical specificity of each case and the patient's safety. Documentation and clinical research are crucial for improving diagnostic criteria and evidence-based surgical choices. Timely identification, precise differentiation from other adnexal masses, and fertility-preserving surgery are essential for managing POCs.
